# Label-Free LC-MS/MS Analysis Reveals Different Proteomic Profiles between Egg Yolks of Silky Fowl and Ordinary Chickens

**DOI:** 10.3390/foods11071035

**Published:** 2022-04-02

**Authors:** Rao Wu, Chen Chen, Xiaoying Zhang

**Affiliations:** 1 Chinese-German Joint Laboratory for Natural Product Research, College of Biological Science and Engineering, Shaanxi University of Technology, Hanzhong 723000, China; 15700351248@163.com; 2 Centre of Molecular & Environmental Biology, Department of Biology, University of Minho, 4710-057 Braga, Portugal; 3 Department of Biomedical Sciences, Ontario Veterinary College, University of Guelph, Guelph, ON N1G 2W1, Canada

**Keywords:** silky fowl (black-bone chicken), egg yolk, label-free proteomic analysis, ovoinhibitor, transthyretin, hemostasis, neuroactive ligand–receptor interaction

## Abstract

The proteomic profiles of Silky fowl egg yolk (SFEY) and Leghorn egg yolk (LEY) were analyzed by bottom-up label-free liquid chromatography–tandem mass spectrometry (LC-MS/MS). From a total of 186 identified proteins, 26 proteins were found significantly differentially abundant between two yolks, of which, 19 were up-regulated and 7 were down-regulated in SFEY, particularly, vitelline membrane outer layer protein 1, transthyretin and ovoinhibitor were up-regulated by 26, 25, and 16 times, respectively. In addition, there were 57 and 6 unique proteins in SFEY and LEY, respectively. Gene Ontology (GO) revealed SFEY contained relatively more abundant protease inhibitors and coagulation-related proteins. Kyoto Encyclopedia of Genes and Genomes (KEGG) analysis revealed differentially abundant proteins in SFEY may be actively involved in the regulation of the neuroactive ligand–receptor interaction pathway. This study provides a theoretical basis for the understanding of proteomic and biological differences between these two yolks and can guide for further exploration of nutritional and biomedical use of Silky fowl egg.

## 1. Introduction

Hen egg is an easily available source of nutritious food. Compared to other animal sourced foods, eggs contain higher protein, lipids, a variety of amino acids and mineral elements [1]. Egg is mainly composed of three parts: eggshell, egg white and egg yolk [2]. Egg yolk represents an important aspect of egg quality and nutritional value, and is the main carrier of egg flavor [3]. The major proteins of egg yolk include serum albumin, ovalbumin, apovitellenin-1, lipovitellins and immunoglobulin Y (IgY) [4].

Proteomic approach enables the identification of different proteins and understanding of protein–protein interactions [5]. Mass spectrometry-based quantitative proteomics has been developed fast and widely used to analyze the structural and functional relationships of various proteins in egg yolk. A comparative proteomic analysis of highland (Tibetan) and lowland egg yolks revealed high antioxidant activity of egg yolk proteins from Tibetan samples [6]. Proteomic analysis of egg yolks with high and low cholesterol contents showed that cholesterol concentration significantly altered yolk protein intensity [7]. Comparative proteomic analysis of different periods of yolk formation revealed changes in the main nutrients in the yolk and helps to understand the formation of the yolk assembly structure [8].

Silky fowls (black-bone chickens) are native to some Asian countries and have been consumed as ethno-remedies, owning to the medicinal properties in enhancing immune system, preventing emaciation and remitting conditions such as menstrual abnormalities and postpartum complications [9,10]. They have been domesticated with a long history, and have formed multiple sub-types, such as Taihe, Yanjin, Wumeng, Muchuan and Lueyang black-bone chickens in China, as well as Thai black-bone chickens [11,12,13]. In addition to the ethno-pharmacological effects of silky fowl meat, the eggs have also showed some properties that are different from ordinary eggs. The Silky fowl eggs are richer in some biological active substances, and are oxidative stable and contain a higher ratio of unsaturated fatty acids [14]. The content of sialic acid (an important molecule in cell–cell interactions) in Silky fowl egg yolk (SFEY) is significantly higher (205.20 mg/egg) than that of ordinary egg yolk (17.77 mg/egg) [15]. Proteomic analysis is a powerful tool to fully exploit the potential value of the eggs. A recent study explored the proteomic differences between egg whites from Silky fowl and ring-necked pheasant, and found that the former has a greater capacity to metabolize cholesterol and is less likely to cause high plasma cholesterol compared to the latter [16]; however, this study did not reference Silky fowl with ordinary commercial chicken, and did not investigate yolks. This study aimed to reveal the proteomic profiles of SFEY through a mass spectrometry-based proteomics approach by comparing SFEY and Leghorn egg yolk (LEY), to further explore the potential nutritional and medical value of this novel egg resource.

## 2. Materials and Method

### 2.1. Preparation of Egg Yolk Samples

Fresh Lueyang Silky fowl eggs and Leghorn eggs were bought from the local farmer market in Hanzhong, China. A total of three eggs in each group were collected as three replicates. Each yolk was carefully washed and separated with deionized water, and the yolk vitelline membrane was carefully removed from the yolk.

### 2.2. Protein Extraction and Digestion

SDT (4% SDS in *w*/*v*, 100 mM Tris-HCl pH 7.6, 100 mM DTT) was used for sample lysis and protein extraction. The protein quantification was performed with the BCA Protein Assay Kit (Bio-Rad, Hercules, CA, USA). Each protein sample (200 μg) was incorporated into 30 μL SDT buffer (4% SDS, 100 mM DTT, 150 mM Tris-HCl pH 8.0) for trypsin digestion by following filter aided proteome preparation (FASP) method [17]. The detergent, DTT and other low-molecular-weight components were removed using UA buffer (8 M Urea, 150 mM Tris-HCl pH 8.0) by repeated ultrafiltration. Then, iodoacetamide (100 μL, 100 mM IAA in UA buffer) was added to block the reduced cysteine residues and the samples were incubated for 30 min in the dark. After the collection of all samples into the ultrafiltration tube, UA (100 μL) was added for centrifugation for 15 min, followed by triplicated operation of adding ammonium bicarbonate (100 μL, 50 mM) and centrifugation for 10 min. Finally, the protein suspensions were digested with trypsin (4 μg; Merck, Darmstadt, Germany) in ammonium bicarbonate buffer (40 μL, 25 mM) overnight at 37 °C, and the resulting peptides were collected as a filtrate. The digest peptides of each sample were desalted on C18 Cartridges (Empore™ SPE Cartridges C18 in standard density, bed I.D. 7 mm, volume 3 mL; Merck, Darmstadt, Germany), concentrated by vacuum centrifugation and reconstituted in formic acid (40 μL, 0.1%, *v*/*v*). The peptide content was estimated by UV light spectral density at 280 nm and calculated on the basis of the frequency of tryptophan and tyrosine in vertebrate proteins.

### 2.3. Liquid Chromatography–Tandem Mass Spectrometry (LC-MS/MS) Analysis

The procedures for LC-MS/MS were similar to a previous description [18]. In detail, each sample was separated by HPLC liquid phase system EasynLC with nano-liter flow rate. The peptides were loaded onto a reverse phase trap column (100 μm 2 cm; Thermo Fisher Scientific, Waltham, MA, USA), connected to the C18-reversed phase analytical column (length: 10 cm, inner diameter: 75 μm) in buffer A (0.1% formic acid) and then separated with a linear gradient of buffer B (84% acetonitrile and 0.1% formic acid) at a flow rate of 300 nL/min controlled by IntelliFlow technology [19]. After chromatographic separation, the samples were analyzed by Q-Exactive mass spectrometer (Thermo Fisher Scientific, Waltham, MA, USA). The mass spectrometer was operated in the form of positive ions, with the scan range of the precursor ion 300–1800 *m*/*z*, and the related parameters as follows: automatic gain control target: 3,000,000, the maximum inject time: 10 ms, dynamic exclusion duration: 40.0 s, resolution of survey scans: 70,000 at *m*/*z* 200, resolution for HCD spectra: 17,500 at *m*/*z* 200, isolation width: 2 *m*/*z*, normalized collision energy: 30 eV and under fill ratio (specifies the minimum percentage of the target value likely to be reached at maximum fill time): 0.1%. The instrument was operated with the use of peptide recognition mode for the collection of 20 fragments after each full scan.

### 2.4. Identification and Quantitation of Proteins

The MS raw data of each sample were combined and searched using the MaxQuant search engine (1.5.3.17) for identification and quantitation analysis [20]. Tandem mass spectra were searched against the NCBI database (UniProt. Gallus gallus. 34,950. 26 October 2020. FASTA). Related parameters and instructions were as follows: cleavage enzyme: trypsin (allowing up to 2 missing cleavages), mass tolerance for precursor ions: 20 ppm in the first search and 6 ppm in the main search, carbamidomethyl on cysteine residues: fixed modification, oxidation on methionine residues: variable modifications and the false discovery rate (FDR): ≤0.01. The same protein can be used for subsequent Perseus analysis if it is identified at least twice in three repeated biological tests. Protein abundance is represented by LFQ (label-free quantitative) value [21], with SFEY/LEY ratio > 2 and *p* value < 0.05 defined as up-regulation in SFEY, ratio < 0.5 and *p* value < 0.05 as down-regulation in SFEY (Table 1).

### 2.5. Bioinformatic Analysis

Perseus 1.3.0.4 software was used to perform statistical analysis on the database search files obtained by MaxQuant. Cluster 3.0 (http://bonsai.hgc.jp/~mdehoon/software/cluster/software.htm (accessed on 6 December 2020)) and Java Treeview software (http://jtreeview.sourceforge.net (accessed on 6 December 2020)) was used to perform hierarchical clustering analysis. The InterProScan software was used to search protein sequences for identifying protein domain signatures from the InterPro member database Pfam [22]. Gene Ontology (GO) [23,24] (http://geneontology.org/ (accessed on 18 December 2020)) and Kyoto Encyclopedia of Genes and Genomes (KEGG) [25] (http://www.kegg.jp/ (accessed on 19 December 2020)) were used to analyze the function and metabolic pathways of differentially abundant proteins. The Fisher’s Exact Test was applied to compare the distribution of each GO classification or KEGG pathway in the differentially abundant proteins and the total identified proteins, and the differential proteins were analyzed for enrichment of GO annotations or KEGG pathway annotations. Based on the STRING database (http://string-db.org/ (accessed on 19 December 2020)), CytoScape 3.2.1 software was used to analyze the interactions between differentially abundant proteins.

### 2.6. Data Availability

The mass spectrometry data have been deposited to the ProteomeXchange Consortium (http://www.proteomexchange.org/ (accessed on 20 March 2022)) via the PRIDE [26] partner repository with the dataset identifier PXD032674. All other data supporting this study are available from the corresponding author on reasonable request.

## 3. Results

### 3.1. Cluster Analysis Showed Obvious Difference of Protein Types and Contents between Two Yolks

**Figure 1:**
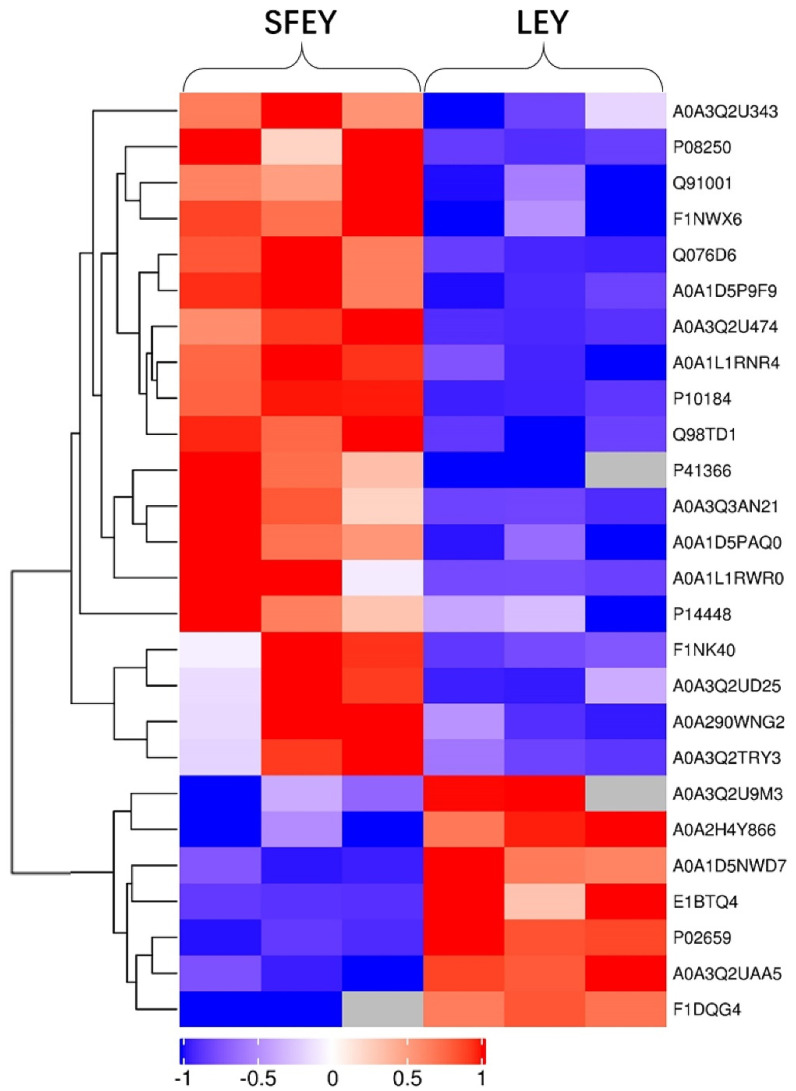
Profiles of differently expressed proteins identified in SFEY and LEY using cluster analysis. A total of 26 proteins were observed with significantly difference. Three biological replicates of SFEY and LEY were presented separately. Red and blue: significantly up-regulated and down-regulated proteins, respectively; gray: no quantitation information.

A total of 1026 peptides and 186 proteins were identified from samples, in which, 143 and 82 proteins were identified in SFEY and LEY, respectively. There were 63 proteins only expressed in one of the yolks, of which, 57 in SFEY and 6 in LEY (Supplementary Table S1, Supplementary Material online). A total of 26 significantly differentially abundant proteins were identified in both yolks, 19 were up-regulated and 7 were down-regulated in SFEY compared to LEY (Table 1). The top five up-regulated proteins in SFEY were vitelline membrane outer layer protein 1, transthyretin, ovoinhibitor, prothrombin and Ig-like domain-containing protein (A0A3Q3AN21). The top five down-regulated proteins in SFEY were Ig-like domain-containing protein (A0A3Q2UAA5), Ig-like domain-containing protein (A0A3Q2U9M3), apovitellenin-1, ovalbumin (Fragment) and complement component 7. The hierarchical cluster analysis displayed in the form of a tree-type heat map visually showed the protein difference of these two yolks (Figure 1). Both SFEY and LEY had good intra-group repeatability. The top ten high-abundant unique proteins in SFEY were inter-alpha inhibitor heavy chain 2, Ig-like domain-containing protein (A0A3Q3AMX7), beta-microseminoprotein-like, ceruloplasmin, coagulation factor X, complement component 8 subunit beta, gallinacin-9, DUF4430 domain-containing protein, coagulation factor XII and angiotensin 1–10. The top unique proteins in LEYs were apolipoprotein B, cilia- and flagella-associated protein 36, dynactin subunit 1, voltage-dependent anion-selective channel protein 1, VWFA domain-containing protein and Ig-like domain-containing protein (A0A3Q2UCM5).

### 3.2. GO Enrichment Analysis Revealed That Peptidase Regulates the Activity of Most Differentially Abundant Proteins Extensively Involved in the Coagulation Process

**Figure 2:**
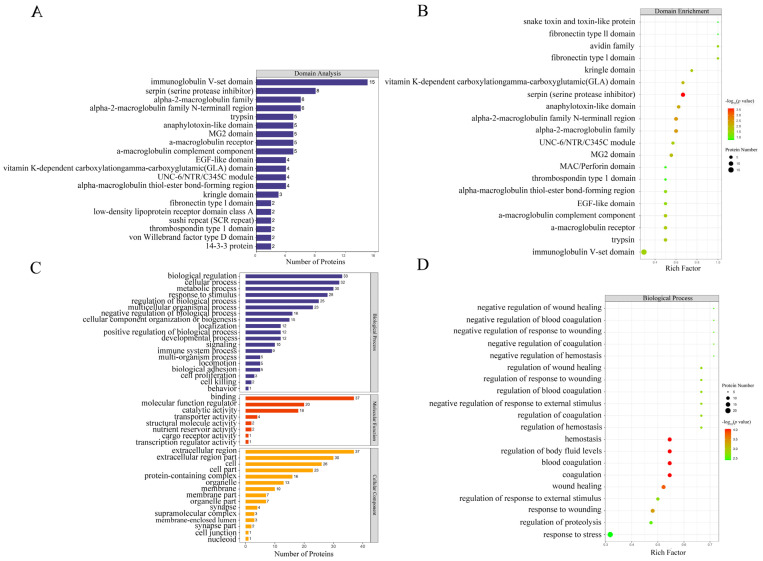

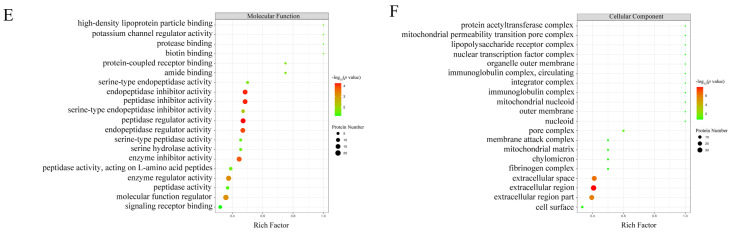
Domain analysis and GO annotation of differentially abundant proteins. Panels (**A**,**C**) represent domain and GO annotation statistics of differentially abundant proteins, respectively. Vertical axis represents domain and GO term; horizontal axis represents the number of differentially abundant proteins corresponding to the domain and GO term. Panel (**B**) represents domain enrichment of differentially abundant proteins. Panels (**D**–**F**) represent GO function enrichment of differentially abundant proteins for biological processes, molecular functions and cellular components, respectively. Vertical axis represents different domain or GO function category; horizontal axis represents the enrichment factor (≤1; numbers of differentially abundant proteins associated with a domain or GO term/numbers of characterized proteins corresponding to the domain and GO term); the colors of the bubble indicate the significance of the enriched domain or GO terms; and the color gradient from green to red represents the increasing significant difference.

Among the proteins with significantly higher or lower abundance, there were four domains dominated: immunoglobulin V-set domain, serpin (serine protease inhibitor), alpha-2-macroglobulin family and alpha-2-macroglobulin family N-terminal region (Figure 2A). The domain enrichment (Figure 2B) indicated that the five domains with the largest enrichment factors were serpin (serine protease inhibitor), alpha-2-macroglobulin family, alpha-2-macroglobulin family N-terminal region, vitamin K-dependent carboxylation/gamma-carboxyglutamic (GLA) domain and kringle domain. However, the immunoglobulin V-set domain had the lowest enrichment level, despite being shared by 15 differentially abundant proteins.

GO annotation (Figure 2C) demonstrated that among those differentially abundant proteins, the top five biological processes were biological regulation, cellular process, metabolic process, response to stimulus and regulation of biological process; the top five molecular functions were binding, molecular function regulator, catalytic activity, transporter activity and structural molecule activity; and the top five cellular components were extracellular region, extracellular region part, cell, cell part and protein-containing complex (Figure 2C). GO enrichment (shown in bubble charts in Figure 2D–F) demonstrated that differentially abundant proteins were enriched for five important biological processes (hemostasis, coagulation, blood coagulation, regulation of body fluid levels and wound healing), five molecular functions (peptidase regulator activity, peptidase inhibitor activity, endopeptidase inhibitor activity, endopeptidase regulator activity and enzyme inhibitor activity) and three cellular components (extracellular region, extracellular space and extracellular region part).

### 3.3. KEGG Pathway Analysis Showed Three Differentially Abundant Proteins (Plasminogen, Prothrombin and Angiotensin 1–10) Associated with Neuroactive Ligand–Receptor Interactions Pathway

**Figure 3:**
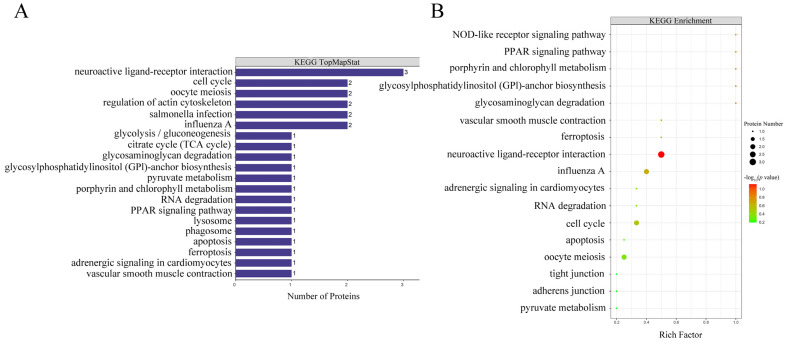
KEGG annotations and pathway enrichment analysis results. Panel (**A**) represents KEGG pathway annotate results of differentially abundant proteins. The vertical axis represents KEGG pathways and the horizontal axis represents the number of differentially abundant proteins involved in each pathway. Panel (**B**) represents KEGG pathway enrichment results of differentially abundant proteins. The vertical axis represents different KEGG pathways and the horizontal axis represents the enrichment factor (≤1; numbers of differentially abundant proteins associated with a KEGG pathway/numbers of characterized proteins corresponding to the pathway). The colors of the bubble indicate the significance of the enriched KEGG pathway, and the color gradient from green to red represents the increasing significant difference.

**Figure 4:**
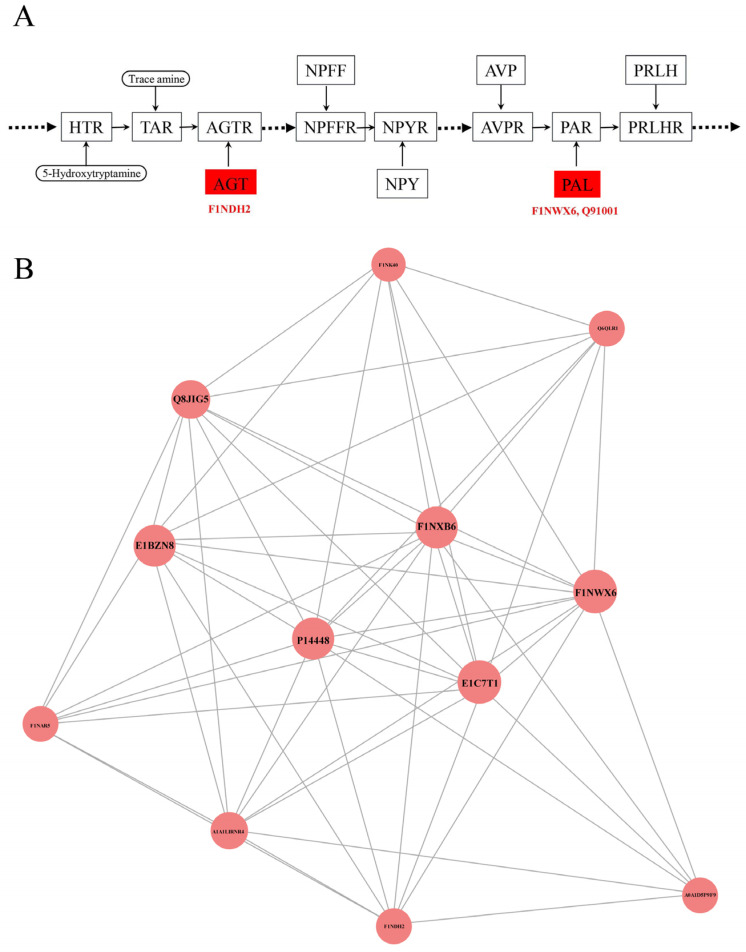
(**A**) Enriched plasminogen, prothrombin and angiotensin 1–10 involved in the metabolic pathway of neuroactive ligand–receptor. The up-regulated proteins in the pathways were summarized. The highly expressed AGT and PARL in SFEY are involved in the metabolic pathway of neuroactive. Square frames represent proteins in the metabolic pathway. Round frames represent small molecular metabolites. The boxes in red indicate the up-regulated proteins. HTR, 5-hydroxytryptamine receptor 2; TAR, trace amine-associated receptor; AGT, angiotensinogen Ⅱ, Ⅲ; AGTR, angiotensin II receptor type 1; NPFF, neuropeptide FF-amide peptide; NPFFR, neuropeptide FF receptor 1; NPY, neuropeptide Y; NPYR, neuropeptide Y receptor; AVP, arginine vasopressin; AVPR, arginine vasopressin receptor 2; PAL, proteinase-activated like; PAR, proteinase-activated receptor; PRLH, prolactin-releasing-like hormone; PRLHR, prolactin-releasing hormone receptor. (**B**) Interaction networks of differentially abundant proteins. Circle nodes: differentially abundant proteins (red: up-regulation; circle size: degree of connectivity of the protein, namely, the number of proteins that directly interact with a protein); lines: protein–protein interactions.

KEGG pathway analysis has been used for systematic and comprehensive exploration of the proteome functions and protein coordination in biological processes, disease mechanisms and drug action mechanisms. KEGG annotation showed that neuroactive ligand–receptor interactions were enriched for the most differentially abundant proteins (F1NWX6, Q91001 and F1NDH2), followed by cell cycle, oocyte meiosis, regulation of actin cytoskeleton, salmonella infection and influenza A (Figure 3A). The KEGG pathway enrichment analysis showed that the *p* values of all pathways were greater than 0.05 and there was no enrichment result in between SFEY and LEY (Figure 3B). In the pathway of the neuroactive ligand–receptor interaction, the expressions of these three proteins (plasminogen, prothrombin and angiotensin 1–10) were up-regulated (Figure 4A).

### 3.4. Protein–Protein Interaction Analysis Showed Strong Functional Relationships among Three Differentially Abundant Proteins (SERPIN Domain-Containing Protein, Fibrinogen Alpha Chain and Plasminogen)

The possible protein–protein interactions of differentially abundant proteins were analyzed based on the STRING database by using the CytoScape software (Figure 4B). Among 26 proteins with significantly different content in both yolks and 63 proteins only expressed in one of the yolks, 12 proteins exhibited protein–protein interactions, in which, 6 proteins (SERPIN domain-containing protein (E1C7T1), fibrinogen alpha chain (P14448), carboxypeptidase B2 (F1NXB6), plasminogen (F1NWX6), α-1-acid glycoprotein (Q8JIG5) and hepatocyte growth factor activator (E1BZN8)) were closely related in function. Furthermore, E1C7T1, P14448 and F1NWX6 demonstrated the strongest interactions.

## 4. Discussion

With the increasing discovery and application of bioactive egg compounds in food and non-food sectors, the comprehensive values of hen egg, particularly the yolk, have been recognized and further explored for both academic and industrial purposes [27]. A classic pilot proteomic study on egg yolk identified 119 proteins grouped into five major categories, including VTG-derived proteins (i.e., phosvitin, apovitellenin-1), proteases and protease inhibitors, vitamin- and cofactor-binding proteins, serum proteins and some proteins from the egg white [4]. With the popularization of proteomic techniques, such as mass spectrometry, increasing studies have investigated on the physiological and biochemical properties of proteins in the egg yolk, the egg white and the entire egg, such as the spoilage properties of eggs under long-term storage [28], changes in yolk proteins in fertilized eggs during the incubation process [29]. However, there are few reports on the differences in the composition and function of proteins in Silky fowl eggs, particularly SFEY, in comparison to the common egg and yolk. Our study attempted to reveal the difference in protein contents between SFEY and LEY, for better understanding and utilization of this edible and medicinal resource. A total of 186 different proteins was identified in samples when combining SFEY and LEY proteomic data, and 143 and 82 proteins were identified in SFEY and LEY, respectively, indicating significantly richer protein content of SFEY. The current study harvested 26 significantly differentially expressed proteins between SFEY and LEY, as well as 63 unique proteins that only exist in one of the yolks.

Eggs contain many proteases and anti-proteases, mainly in egg whites [30]. However, in our study, significantly higher abundance of proteases and their inhibitors were detected in SFEY (Table 2). Based on different domains, α-2-macroglobulin family or serpin (serine protease inhibitor), these protease inhibitors are divided into two groups. The proteins containing the domain of alpha-2-macroglobulin family include alpha-2-macroglobulin-like 4, C4a anaphylatoxin (Q07606, A0A290WNG2), complement component 3 (C3) and complement component 5 (C5) (Table 2). Alpha-2-macroglobulin is a broad spectral protease inhibitor, presented in the plasma of all vertebrates [31]. Anaphylatoxin is a substance derived from complement activation that causes smooth muscle contraction, capillary leakage and even anaphylactic shock [32]. C4a anaphylatoxin, C3 and C5 are all derived from the blood complement system, which participate in the body’s immune response [32]. The proteins containing serpin (serine protease inhibitors) mostly include SERPIN domain-containing protein (A0A1D5PI58, E1C7T1 and F1NAR5), ovalbumin-related protein Y (Table 2), which belong to serine protease inhibitors and may be involved in cell proliferation, tissue reconstruction and physiology process related to follicle formation, extra-embryonic structure development, eggshell bio-mineralization, egg defense and embryo nutrition [33]. Inter-alpha inhibitor heavy chain 2, a subunit of inter-alpha inhibitor, is structurally related to serine protease inhibitors. It is a natural brain tumor invasive factor and has been used as a potential indicator of tumor malignancy [34]. However, the function of this protein from chicken is not known. This protein is worth further attention as it is the most abundant unique proteins in SFEY (Supplementary Table S1). In addition, ovoinhibitor also belongs to the serine protease inhibitors, a protein that plays an important role in the antimicrobial defense of eggs against *Bacillus* spp., preventing contamination of edible eggs (non-fertilized eggs) and protecting chicken embryos (fertilized eggs) [35]. Furthermore, anti-proteases are thought to be involved in antimicrobial defense primarily by inhibiting bacterial proteases secreted by pathogens during host colonization [30]. A comparative proteomic study of egg yolk during storage found that increased degradation of protease inhibitors (such as ovoinhibitor and α-2-macroglobulin-like protein) along with the prolonged storage may lead to imbalance of protease and anti-protease in yolk, which may play a key role in the degradation of egg yolk proteins [28]. Vitelline outer layer protein 1 binds tightly with ovomucin through lysozyme and vitelline outer layer protein 2 and participates in the formation of the vitelline membrane [36], which separates the egg yolk from the white and functions as the indispensable antibacterial barriers for poultry eggs [37]. Meanwhile, ovoinhibitor is a natural antioxidant protein in the yolk together with ovalbumin, lysosome, phosphoprotein and phospholipids, etc. [38], they can regulate the production and loss of reactive oxygen, such as hydrogen peroxide (H_2_O_2_) and hydroxyl radical (•OH), maintain the body’s redox homeostasis and protect the body from oxidative stress [38]. Ceruloplasmin also has a similar antioxidant effect by consisting of a copper-containing ferrous oxidase that protects tissues from iron-mediated free radical damage by oxidizing toxic ferrous iron to non-toxic form [39]. In addition, the egg yolk might have an antithrombotic effect by inhibiting platelet aggregation and fibrin formation in humans [40]. In our study, abundant proteases (antithrombin-III, heparin cofactor II, and kininogen-1, etc.; Table 2), which are mainly involved in the coagulation process, presented in SFEY, as analyzed by GO function. Antithrombin III and heparin cofactor II are the main regulators of blood flow, the former mainly inhibits factor Xa and thrombin and the latter exclusively inhibits thrombin [41]. Kininogen-1 has anti-angiogenic effects and involved in many pathological processes, including fibrinolysis, thrombosis and inflammation [42]. The protein–protein interactions (Figure 4B) further indicated that some significantly abundant proteins, represented by coagulation factor XII, plasminogen and fibrinogen α-chain, are involved in hemostasis, suggesting that the consumption of SFEY may have a positive effect on blood flow regulation.

Apart from antibacterial and antioxidant properties, it is interesting to explore the potential neurological values of SFEY. KEGG analysis showed that three proteins, prothrombin, plasminogen and angiotensin 1–10, significantly regulate neural active ligand–receptor interaction pathways, which is directly related to neural functions, as these proteins can bind with the intracellular receptors, which further activate the transcription factors and to regulate the downstream gene expression [43]. As mentioned earlier, prothrombin and plasminogen participate in the regulation of neurovascular and body fluid levels. Angiotensin (AT) binds to AT1 and AT2 receptors in the central nervous system and regulates vasoconstriction in the peripheral circulation [44], which is consistent with the KEGG result (Figure 3). Furthermore, a recent animal study demonstrated that neurons were abundant and congested in the rat hippocampus after an egg yolk diet, indicating egg yolk’s beneficial effect on brain development and health [45], despite the mechanism and composition basis behind this finding remains unknown. The higher abundant proteins in SFEY related to the neuroactive ligand–receptor interaction (Figure 4A) may provide clues for further investigation. Another observation supporting the beneficial effects of egg diet on neurodevelopment is transthyretin, which is abundant and up-regulated 24 times in SFEY. This a highly conserved homotetrameric protein mainly synthesized by the liver and the choroid plexus of the brain. In addition to its function as a major carrier of thyroxine and retinol, transthyretin is an important protein in maintaining the physiology of the peripheral and central nervous system, namely through its involvement in behavior, maintenance of normal cognitive processes during aging, amidating neuropeptide processing and neuro regeneration [46]. Furthermore, transthyretin has a neuroprotective effect in Alzheimer’s disease [47,48], and the plasma levels of this protein can be used as a biomarker for osteoporosis in elderly subjects [49]. It is therefore worthwhile to further explore the “brain-supportive” effect of SFEY diets.

Eggs are rich in cholesterol, approximately 213 mg per egg, mainly in the yolk [50]. Clinical studies have shown that more cholesterol was detected in blood samples from women consuming whole eggs compared to a yolk-free diet [51]. High cholesterol levels make some people tend to limit the consumption of eggs, particularly yolks. However, it has been shown that there is active lipid and cholesterol metabolism in Silky egg whites [16], our study observed significant differences in apovitellenin-1 and Apolipoprotein A-I between SFEY and LEY (Table 1). Apolipoprotein A-I, as a part of high-density lipoprotein, is involved in the reverse cholesterol transport process in chicken [52]. Meanwhile, a previous study confirmed that avian apolipoprotein A-I is involved in the local transport of lipids in myelin biosynthesis [53]. It is interesting to observe that there were obvious differences in the composition of lipid-binding proteins between SFEY and LEY. Contrary to a significant up-regulation of apolipoprotein A-I, apolipoprotein B and apovitellenin-1 were both down-regulated in SFEY, which are involved in the compositions of very low-density lipoproteins related to the accumulation of lipid and cholesterol in the yolk [54]. These results indicate that SFEY may be a good dietary source with low cholesterol.

## 5. Conclusions

Obvious proteomic differences have been observed between SFEY and LEY. A total of 143 and 82 proteins were identified in these two yolks, respectively. Among the shared proteins, 26 showed significant differences: 19 were up-regulated and 7 were down-regulated. In addition, there were 57 and 6 kinds of unique proteins in SFEY and LEY, respectively. Some differently expressed proteins, such as transthyretin, ovoinhibitor, ceruloplasmin and inter-alpha inhibitor heavy chain 2, are worthy of further explorations for their nutritional and biomedical values.

## Figures and Tables

**Table 1:** List of significantly differentially abundant proteins in SFEY versus LEY.

Protein Name	Accessions	Unique Peptides	SFEY/LEY Fold Changes	*p* Value	GO Terms
Vitelline membrane outer layer protein 1	P41366	1	26.452 ↑	0.007	Extracellular region
Transthyretin	A0A1L1RWR0	3	24.708 ↑	0.023	Cellular lipid metabolic process; hormone metabolic process
Alpha-2-macroglobulin-like 4	F1NK40 H1AC38	30	17.041 ↑	0.027	Molecular function regulator; enzyme regulator activity
Ovoinhibitor	P10184	18	15.797 ↑	<0.001	Regulation of proteolysis; peptidase regulator activity; serine-type endopeptidase inhibitor activity
Ig-like domain-containing protein	A0A3Q3AN21; A0A3Q2UGI5; A0A3Q2TZD6; A2N890; A2N888	2	11.154 ↑	0.009	Immune system process
C4a anaphylatoxin	Q076D6; D4QB02; D4QB00	2	8.493 ↑	<0.001	Response to stress; proteolysis; peptidase regulator activity
Apolipoprotein A-I	P08250	10	7.843 ↑	0.006	Response to stress; enzyme regulator activity
Ig-like domain-containing protein	A0A3Q2U474	3	7.608 ↑	<0.001	Immune system process
Ig-like domain-containing protein	A0A1D5PAQ0; A0A3Q2UGD4; A0A3Q2U2P6	2	6.610 ↑	0.003	Immune system process
Avidin-related protein 6-like	A0A3Q2UD25	5	5.915 ↑	0.044	Amide binding; biotin binding
Complement component 3	A0A1D5P9F9; A0A1D5PWR4; A0A1D5PFG3; A0A1D5PQ85; A6N9E0; Q2MV09; Q6PPA9	10	5.416 ↑	<0.001	Regulation of response to external stimulus; regulation of proteolysis; peptidase regulator activity; endopeptidase regulator activity
Kininogen 1	A0A1L1RNR4	5	5.060 ↑	<0.001	Hemostasis; blood coagulation; peptidase regulator activity
Complement factor H	A0A3Q2TRY3A0A3 Q2TXN4E1C7P4 A0A3Q3AX54 A0A3Q3AK26	25	4.301 ↑	0.042	Immune effector process; protein activation cascade
C4a anaphylatoxin	A0A290WNG2	3	3.694 ↑	0.035	Response to stress; proteolysis; peptidase regulator activity
Prothrombin	Q91001; F1NXV6	11	3.534 ↑	0.006	Hemostasis; blood coagulation; serine-type endopeptidase activity
Ig-like domain-containing protein	A0A3Q2U343	1	2.831 ↑	0.018	Immune system process
Plasminogen	F1NWX6; Q7LZF3	24	2.816 ↑	0.002	Hemostasis; blood coagulation; serine-type endopeptidase activity
PIT 54	Q98TD1; A0A1L1S0P1	13	2.651 ↑	<0.001	Scavenger receptor activity
Fibrinogen alpha chain	P14448; F1P4V1	13	2.527 ↑	0.043	Hemostasis; blood coagulation; response to stress
Ig-like domain-containing protein	A0A3Q2UAA5	7	0.413 ↓	<0.001	Immune system process
Dishevelled binding antagonist of beta catenin 2	A0A1D5NWD7	1	0.380 ↓	0.002	Negative regulation of cell adhesion; regulation of response to stimulus; tube development
Ig-like domain-containing protein	A0A3Q2U9M3	2	0.373 ↓	0.008	Immune system process
Apovitellenin-1	P02659	7	0.248 ↓	<0.001	Lipid metabolic process; enzyme inhibitor activity
Ovalbumin (Fragment)	A0A2H4Y866; A0A2H4Y8E8; P0102; A0A2H4Y8S8; A0A2H4Y8R9; A0A2H4Y8R6; A0A2H4Y8R1	6	0.233 ↓	0.001	Extracellular region
Complement component 7	F1DQG4; E1C6U2	17	0.19 ↓	<0.001	Immune effector process; protein activation cascade
Avidin	E1BTQ4; A0A3Q2U4N2; A0A3Q2UC51	3	0.113 ↓	0.003	Amide binding; biotin binding

↑, up-regulation; ↓, down-regulation.

**Table 2:** List of domains and GO categories contained in Proteins^Var^.

Domain Name	GO Category	Proteins^Var^ Number	Proteins^Var^ Name
Serpin (serine protease inhibitor)	/	8	SERPIN domain-containing protein (A0A1D5PI58, E1C7T1, F1NAR5), Heparin cofactor II (Fragment), Ovalbumin-related protein Y, Angiotensin 1–10, Antithrombin-III (Fragment), Ovalbumin (A0A1D5PI58)
Alpha-2-macroglobulin family	/	6	Alpha-2-macroglobulin-like 4, C4a anaphylatoxin (Q07606, A0A290WNG2), Complement component 3, Complement component 5, Uncharacterized protein (A0A3Q3B2L3)
Alpha-2-macroglobulin family N-terminal region	/	6	Alpha-2-macroglobulin-like 4, C4a anaphylatoxin (Q07606, A0A290WNG2), Complement component 3, Complement component 5, Uncharacterized protein (A0A3Q3B2L3)
Anaphylotoxin-like domain	/	5	C4a anaphylatoxin (Q07606, A0A290WNG2), Complement component 3, Complement component 5, Fibulin-1
Vitamin K-dependent carboxylation/ gamma-carboxyglutamic (GLA) domain	/	4	Prothrombin, Vitamin K-dependent protein S, Anticoagulant protein C, Coagulation factor X
MG2 domain	/	5	Alpha-2-macroglobulin-like 4, C4a anaphylatoxin (Q07606, A0A290WNG2), Complement component 3, Complement component 5
Kringle domain	/	3	Prothrombin, Plasminogen, Coagulation factor XII
Trypsin	/	5	Prothrombin, Plasminogen, Coagulation factor XII, Anticoagulant protein C, Coagulation factor X
A-macroglobulin receptor	/	5	Alpha-2-macroglobulin-like 4, C4a anaphylatoxin (Q07606, A0A290WNG2), Complement component 3, Complement component 5
A-macroglobulin complement component	/	5	Alpha-2-macroglobulin-like 4, C4a anaphylatoxin (Q07606, A0A290WNG2), Complement component 3, Complement component 5
/	Peptidase regulator activity, peptidase inhibitor activity, endopeptidase inhibitor activity, endopeptidase regulator activity, enzyme inhibitor activity.	15	SERPIN domain-containing protein (E1C7T1, F1NAR5), Heparin cofactor II (Fragment), Ovalbumin-related protein Y, Angiotensin 1–10, Antithrombin-III (Fragment), Ovoinhibitor, Alpha-2-macroglobulin-like 4, C4a anaphylatoxin (Q07606, A0A290WNG2), Complement component 3, Complement component 5, Kininogen-1, Inter-alpha inhibitor heavy chain 2, Uncharacterized protein (A0A3Q3B2L3)
/	Serine-type endopeptidase activity.	5	Prothrombin, Plasminogen, Coagulation factor XII, Anticoagulant protein C, Coagulation factor X
/	Hemostasis, coagulation, blood coagulation, regulation of body fluid levels, wound healing, response to wounding.	12	Prothrombin, Plasminogen, Coagulation factor XII, Anticoagulant protein C, Coagulation factor X, Vitamin K-dependent protein S, Fibrinogen alpha chain, Kininogen-1, Peptidase_M14 domain-containing protein, SERPIN domain-containing protein (F1NAR5), Antithrombin-III (Fragment), Heparin cofactor II (Fragment)
/	Regulation of response to wounding, regulation of wound healing, regulation of blood coagulation, regulation of coagulation, regulation of hemostasis	6	Kininogen-1, Vitamin K-dependent protein S, Plasminogen, Peptidase_M14 domain-containing protein, SERPIN domain-containing protein (F1NAR5), Antithrombin-III (Fragment)
/	Extracellular region, extracellular space, extracellular region part	30	SERPIN domain-containing protein (A0A1D5PI58, E1C7T1, F1NAR5), Heparin cofactor II (Fragment), Ovalbumin-related protein Y, Angiotensin 1–10, Antithrombin-III (Fragment), Ovalbumin (A0A1D5PI58), Alpha-2-macroglobulin-like 4, C4a anaphylatoxin (Q07606, A0A290WNG2), Complement component 3, Complement component 5, Fibrinogen alpha chain, 60 kDa heat shock protein, Apovitellenin-1, Apolipoprotein A-I, Gallinacin-9, Ceruloplasmin, Glycosyl-phosphatidylinositol-specific phospholipase D, Kininogen-1, Transthyretin, Alpha-1-acid glycoprotein, Coagulation factor XII, Peptidase_M14 domain-containing protein, Ovoinhibitor, Coagulation factor X, 14-3-3 protein zeta, Vitelline membrane outer layer protein 1

Proteins^Var^, unique and differently expressed proteins in two yolks.

## Data Availability

The mass spectrometry data have been deposited to the ProteomeXchange Consortium (http://www.proteomexchange.org/ (accessed on 20 March 2022)) via the PRIDE partner repository with the dataset identifier PXD032674. All other data supporting this study are available from the corresponding author on reasonable request.

## Supplementary Materials

The following supporting information can be downloaded at: https://www.mdpi.com/article/10.3390/foods11071035/s1, Table S1: List of unique proteins in silky fowl and ordinary egg yolks.

## Abbreviations

SFEY	Silky fowl egg yolk
LEY	Leghorn egg yolk
HTR	5-hydroxytryptamine receptor 2
TAR	Trace amine-associated receptor
AGT	Angiotensinogen II, III
AGTR	Angiotensin II receptor type 1
NPFF	Neuropeptide FF-amide peptide
NPFFR	Neuropeptide FF receptor 1
NPY	Neuropeptide Y
NPYR	Neuropeptide Y receptor
AVP	Arginine vasopressin
AVPR	Arginine vasopressin receptor 2
PAL	Proteinase-activated like
PAR	Proteinase-activated receptor
PRLH	Prolactin-releasing-like hormone
PRLHR	Prolactin-releasing hormone receptor.
