# Supplementary material for: Label-Free LC-MS/MS Analysis Reveals Different Proteomic Profiles between Egg Yolks of Silky Fowl and Ordinary Chickens
Source: Foods. 2022 Apr 2;11(7):1035. doi: 10.3390/foods11071035 (PMC8997978; doi:10.3390/foods11071035)
Supplement: Supplementary file 1 [file foods-11-01035-s001.zip › foods-1628756-supplementary.pdf]

Table S1 List of unique proteins in silky fowl and ordinary egg yolks

Protein names	Accessions	Unique peptides	Source of specific proteins	GO terms
Inter-alpha inhibitor heavy chain 2	B3VE14; F1NIU3	9	√	Glycosamin glycan metabolic process; peptidase regulator activity; peptidase inhibitor activity
Ig-like domain-containing protein	A0A3Q3AMX7	2	√	Immune system process
Beta-microseminoprotein-like	A0A3Q2U3V9	2	√	Undefined★
Ceruloplasmin	A0A3Q3B296; A0A1D5PBP6	12	√	Cation transport; ferroxidase activity
Coagulation factor X	P25155	5	√	Hemostasis; coagulation; serine-type endopeptidase activity
Complement component 8 subunit beta	E1C7C1	9	√	Regulation of response to stimulus; immune effector process
Gallinacin-9	Q6QLR1; A0A0N9WLX3	1	√	Response to stress; G protein-coupled receptor binding
DUF4430 domain-containing protein	A0A3Q2U8Y5	2	√	Vitamin transport; response to stimulus; cobalamin binding
Coagulation factor XII	E1BZN8	4	√	Hemostasis; blood coagulation; serine-type endopeptidase activity
Angiotensin 1-10	F1NDH2	1	√	Regulation of proteolysis; peptidase regulator activity; serine-type endopeptidase inhibitor activity
SERPIN domain-containing protein	F1NAR5	1	√	Hemostasis; blood coagulation; peptidase regulator activity; serine-type endopeptidase inhibitor activity
IgGFc-binding protein-like	A0A1D5P6F4	10	√	Undefined★
Ensconsin	F1NYS2;	1	√	Microtubule

	A0A3Q2U3B6; Q5ZIA2; A0A3Q2UAA8; A0A3Q2TSC0; A0A1D5PR58			cytoskeleton organization
Complement component 5	A0A1D5PD98; E1BRS7; A0A1D5PC67	20	√	Immune effector process; protein activation cascade
Heparin cofactor II (Fragment)	O73840; A0A1D5PLZ2	1	√	Hemostasis; blood coagulation; peptidase regulator activity; serine-type endopeptidase inhibitor activity
Ig-like domain-containing protein	A0A3Q3AKX3;A0A3Q2TTN1	1	√	Immune system process
CN hydrolase domain-containing protein	A0A1D5PEU7; E1BUA6	1	√	Nitrogen compound metabolic process; hydrolase activity
Ig-like domain-containing protein	A0A3Q2TY60	1	√	Immune system process
Protein piccolo (Fragment)	Q9PU36; A0A1D5PRV2; A0A3Q2U9D6	3	√	Multicellular organismal process; synapse organization; calcium ion binding
Ig-like domain-containing protein	A0A3Q2TXP7; A0A3Q2TYY3	1	√	Immune system process
Vitellogenin-2	P02845;Q6BCB8	1	√	Response to stimulus; nutrient reservoir activity
UPAR/Ly6 domain-containing protein	A0A1L1RYU0	3	√	Regulation of response to stimulus; signaling receptor binding
Ovalbumin-related protein Y	P01014; I0J178; E1BTF4; I0J179	4	√	Regulation of proteolysis; peptidase regulator activity; serine-type endopeptidase inhibitor activity
SERPIN domain-containing protein	E1C7T1	5	√	Regulation of proteolysis; peptidase regulator activity
Fibronectin	A0A3Q2TW07; A0A1D5NU50;	28	√	Extracellular region

	F1NJT3; F1NJT4; P11722; O57403			
Apo AI promoter B-region binding protein (Fragment)	Q9DE41;F1NHT5	2	√	Response to stress; negative regulation of response to external stimulus; enzyme regulator activity
Thrombospondin-3 (Fragment)	Q8QGY2	1	√	Cell adhesion; calcium ion binding
Ig-like domain-containing protein	A0A3Q2TW03	0	√	Immune system process
Attractin	A0A3Q2UQ06; F1P2W2	2	√	Integral component of membrane; signaling receptor activity
60 kDa heat shock protein, mitochondrial	Q5ZL72	10	√	Regulation of proteolysis; response to stress; protein-lipid complex binding
Ig-like domain-containing protein	A0A3Q2UH03	1	√	Immune system process
Transcription factor SOX-2	P48430	1	√	Negative regulation of cellular metabolic process
Ig-like domain-containing protein	A0A3Q2TYH6	2	√	Immune system process
Alpha-1-acid glycoprotein	Q8JIG5; A7UEB0	2	√	Regulation of immune system process; immune system process
14-3-3 protein epsilon	Q5ZMT0	5	√	Response to stress; molecular function regulator
Anticoagulant protein C	Q804X5; F1NGY6	2	√	Hemostasis; blood coagulation; serine-type endopeptidase activity
SERPIN domain-containing protein	A0A1D5PI58; R9TNA6; P01013	4	√	Extracellular region; serine-type endopeptidase inhibitor activity
Polyubiquitin (Fragment)	Q9PST8; P79781; Q91021; A0A1D5P546; P0CG62; Q91022; O42388; A0A1L1RTM7	3	√	Regulation of proteolysis

Antithrombin-III (Fragment)	Q91422; F1NLP7	2	√	Hemostasis; peptidase regulator activity; serine-type endopeptidase inhibitor activity
Fibulin-1	O73775; F1NX60; A0A1L1RU28	5	√	Extracellular structure organization; peptidase regulator activity
Ig-like domain-containing protein	A0A3Q2U5V5	2	√	Immune system process
Ig-like domain-containing protein	A0A3Q2U3K4	1	√	Immune system process
Tubulin alpha chain	A0A1D5NW27; P02552; F1NWX0; F1NW97; A0A3Q2U7S4	0	√	Regulation of biological quality; synapse organization
Ubiquitinyl hydrolase 1	V9GVG9; E1BWJ5	2	√	Proteolysis; peptidase activity, acting on L-amino acid peptides
WH1 domain-containing protein	A0A3Q3A4T5	1	√	Catalytic activity, acting on a protein
N-acetylglucosamine-6-sulfatase	F1NI04	2	√	Glycosaminoglycan metabolic process
Peptidase_M14 domain-containing protein	F1NXB6	5	√	Hemostasis; blood coagulation
Pyruvate dehydrogenase E1 component subunit alpha	Q5F426; A0A1D5PEH3	2	√	Acetyl-CoA biosynthetic process
Actin, cytoplasmic 1	P60706; P53478; P63270; P68139; P68034; P08023; F1P476; A0A1D5NV17; A0A3Q3AWV3; A0A1D5PK53; Q90736; B5M200; A0A3Q3A5C8; Q0QWA1; G8HUH5; A9YXX3;	1	√	Multicellular organismal process; structural constituent of postsynaptic actin cytoskeleton

	A9YXX2; G4WW42; E1BXV2			
Glycosyl-phosphatidylinositol-specific phospholipase D	A0A1D5PHH6; A0A3Q2ULQ6	2	√	Glycerolipid metabolic process; glycosylphosphatidylinositol phospholipase D activity
Glutamatedehydrogenase	A0A2L2FP59; A0A1D5NT61; P00368	2	√	Nitrogen compound metabolic process; oxidoreductase activity, acting on the CH-NH2 group of donors
Vitamin K-dependent protein S	A0A3Q2U504; E1C6L4	12	√	Hemostasis; blood coagulation; response to stress
Eukaryotic translation elongation factor 1 alpha 1	A0A1I7Q425; A0A1D5P5X9; Q90835; Q5ZKM2; A0A1L1RRR1	1	√	GTPase activity; purine ribonucleoside binding
14-3-3 protein zeta	Q5ZKC9; A0A1L1RSI2	6	√	Extracellular region
Apolipoprotein B	Q197X2; P11682; Q7LZ77	6	×	Lipid transporter activity
Cilia- and flagella-associated protein 36	A0A3Q2U353; A0A1D5PW31; A0A3Q2U0Q7	1	×	
Dynactin subunit 1	P35458; A0A1D5PEZ3; A0A1D5PWC0; A0A1D5PVT2; A0A3Q3APS1; A0A1D5PCY8; A0A3Q2UPN5; A0A1D5NTW5	3	×	Regulation of intracellular protein transport; microtubule end
Voltage-dependent anion-selective channel protein 1	A0A1D5NTT4; E1BYN7	3	×	Response to stress; negative regulation of metabolic process
VWFA domain-containing protein	E1BZM3	1	×	RNA 3'-end processing; snRNA metabolic process
Ig-like domain-containing	A0A3Q2UCM5	1	×	Immune system process

protein

Notes: √, unique proteins in SFEYs; ×, unique proteins in LEYs; ★, the protein has no defined GO function. All proteins are arranged in descending order of abundance.
